# Editorials

**Published:** 1912-05

**Authors:** 


### EDITORIAL

The Business Office of the *Journal-Record* is 1 1-2 to 5 1-2 South Broad Street.
The Editorial Office is 1014-15 Century Building.
Address all Business Communications to *Journal-Record of Medicine*, 1 1-2 to 5 1-2 South Broad Street.
Make remittances payable *to The Journal-Record of Medicine.*

On matters pertaining to the Editorial and Original Communications, address Edgar G. Ballenger, M. D., Atlanta, Ga.
Reprints of Original Articles will be furnished at cost price. Requests for the same should always be made in THE MANUSCRIPT.

We will present, postpaid, on request, to each contributor of an original article, twenty (20) marked copies of *The Journal-Record of Medicine* containing such article.

### NEOSALVARSAN—"914."

Prof. Ehrlich, in his further indefatigable work, has found in preparation nine hundred and fourteen an improvement on salvarsan. It is said to be a "condensation of formaldehydesulphoxyl acid sodium with salvarsan." It looks like salvarsan and in solution smells like it, but is neutral in reaction and is readily soluble in water. No preparation is required except to dissolve it in freshly distilled water. Aside from this advantage over salvarsan, neosalvarsan, as it is called, causes much less systemic reaction after its administration; the patients stand it much better, therefore it is given in larger doses, which may be repeated more frequenty. On account of these facts, it seems that neosalvarsan will prove more effective and less disagreeable than was salvarsan.

Through the courtesy of Prof. Ehrlich, I was sent a supply of neosalvarsan April the 10th, and from the 124 injections so far given, I am in position to verify the claims so far made for this preparation. In fact, from the larger doses repeated at shorter intervals it has seemed that the various syphilitic lesions have healed even more promptly than they did after salvarsan. The patients who have taken both remedies express a decided preference for neosalvarsan. The much milder reaction seems also to make it safer so that, in all probability, it will have fewer contraindications. Schreiber, of Madgeburg, Germany, has administered 1200 injections to 230 patients since last October, and is very enthusiastic in his praise of its advantages. It is not only a very effective remedy, but Schreiber has seen no neurorecurrences following its administration.

Its potency as a spirochaetocide has been amply demonstrated to us by the prompt manner in which the germs disappeared from chancres in which they were very abundant at the time of the treatment. Syphilitic manifestations, such as the initial lesion, skin rash, mucous patches, sore throat, syphilitic rheumatism, periostitis, and many minor complaints, have quickly healed under the neosalvarsan treatment. We have given 122 of these treatments intravenously, and 2 intramuscularly to a baby.

The average doses given are about as follows: 0.9 gramme for men and 0.75 for women, repeated every two or three days until three or four doses are taken. For infants and children, the doses range from 0.05 gramme to 0.15 gramme.

In our own work we have recommended further that at least two more injections be taken at the end of a month and perhaps one more treatment 30 days later, hoping thus to remove, if possible, the likelihood of a relapse, and at the same time eliminating, we hope, the use of mercury—at least until it is clearly indicated. To give it indiscriminately would confuse the situation and make our results appear better than they really are. If a course of injections of salvarsan or neosalvarsan fail we think, unless a further aplpication of it cures all lesions and renders the blood test negative, that at least a two-year course of mercury should be advised.

Judging from our experience with 678 injections of salvarsan and neosalvarsan during the past 18 months, the patients who receive adequate doses at properly spaced intervals rarely need mercury to supplement the treatment. There is plenty of time to give it after it is found to be necessary, and then it does not mask symptoms that would otherwise demand more active treatment.

EDGAR G. BALLENGER, M. D.

### CERTIFICATES OF HEALTH, MINISTERS AN MARRIAGE.

The stand taken recently by a minister in Chicago, who declared he would heneceforth demand a certificate of health from a reputable physician before he would perform the marriage ceremony, should open the way for a crusade that could do an immense amount of good.

We all realize full well the futility of attempts at passing legal measures to demand a certificate of health before marriage, and yet we know equally well the necessity of some such measure to lessen the innocent suffering and disease of unsuspecting brides. The trouble with legal measures is to find rules that would determine who is "fit" and who is "unfit." The wide degree of latitude offered physicians and ministers would be the commendable feature of such a plan as the one suggested. Those in good health would no more mind the examination than they would the one demanded by insurance companies. Only the diseased could object and it would probably make them more careful than they are at present.

While the deterrent effect might not be as great as desired, the educational feature would more than make up for any other shortcomings the method might have. Mothers, fathers, brides and grooms would all concern themselves as to "fitness" and good health and would soon realize certain dangers that are not now dreamed of.

While many would avail themselves of justices of the peace, the evasion of the medical examination would soon arouse suspicion and make such marriages unpopular. The cities and states could well afford to provide means whereby those unable to pay for an examination could have it done by the regular city or county physicians. No reasonable person could say that the romance of marriage would be affected by such a certificate *demanded for all.* It would not cast a reflection upon either party. If the physicions of the country, who realize more fully than any other class of citizens the need of premarital certificates of health would urge the ministers to make such a demand, we would soon have a state of affairs far better than legal measure could ever provide.

